# Prioritizing the Mental Health and Well-Being of Healthcare Workers: An Urgent Global Public Health Priority

**DOI:** 10.3389/fpubh.2021.679397

**Published:** 2021-05-07

**Authors:** Lene E. Søvold, John A. Naslund, Antonis A. Kousoulis, Shekhar Saxena, M. Walid Qoronfleh, Christoffel Grobler, Lars Münter

**Affiliations:** ^1^Independent Researcher, Oslo, Norway; ^2^Department of Global Health and Social Medicine and Harvard Medical School, Boston, MA, United States; ^3^Mental Health Foundation, London, United Kingdom; ^4^Department of Global Health and Population, School of Public Health, Harvard University, Boston, MA, United States; ^5^21HealthStreet, London, United Kingdom; ^6^Faculty of Health Sciences, School of Health Systems and Public Health, University of Pretoria, Pretoria, South Africa; ^7^Danish Committtee for Health Education, Copenhagen, Denmark

**Keywords:** mental health, healthcare workers, COVID-19, public health emergencies, burnout, self-care, psychological interventions, healthcare policies

## Abstract

The COVID-19 pandemic has had an unprecedented impact on health systems in most countries, and in particular, on the mental health and well-being of health workers on the frontlines of pandemic response efforts. The purpose of this article is to provide an evidence-based overview of the adverse mental health impacts on healthcare workers during times of crisis and other challenging working conditions and to highlight the importance of prioritizing and protecting the mental health and well-being of the healthcare workforce, particularly in the context of the COVID-19 pandemic. First, we provide a broad overview of the elevated risk of stress, burnout, moral injury, depression, trauma, and other mental health challenges among healthcare workers. Second, we consider how public health emergencies exacerbate these concerns, as reflected in emerging research on the negative mental health impacts of the COVID-19 pandemic on healthcare workers. Further, we consider potential approaches for overcoming these threats to mental health by exploring the value of practicing self-care strategies, and implementing evidence based interventions and organizational measures to help protect and support the mental health and well-being of the healthcare workforce. Lastly, we highlight systemic changes to empower healthcare workers and protect their mental health and well-being in the long run, and propose policy recommendations to guide healthcare leaders and health systems in this endeavor. This paper acknowledges the stressors, burdens, and psychological needs of the healthcare workforce across health systems and disciplines, and calls for renewed efforts to mitigate these challenges among those working on the frontlines during public health emergencies such as the COVID-19 pandemic.

## Introduction

With the emergence of the coronavirus disease (COVID-19) pandemic in late 2019, and the World Health Organization declaring it a global pandemic on 11th March 2020, health systems in many countries have been at times overwhelmed and stretched past their limits in terms of capacity and resources while striving toward continued delivery of quality care. The challenges for health systems, further complicated by the emergence of new more infectious variants of the virus, are likely to persist—even though infection rates have decreased in many parts of the world and the vaccine roll out progresses at a rapid pace at the time of writing this article—because we are now facing a second and equally serious pandemic of mental health challenges. The threats to mental health run deep within communities and are far reaching, affecting the millions of individuals who have been traumatized during national or regional lockdowns, left vulnerable to substance use or loneliness, those who have lost loved ones to the virus or face heightened anxieties of getting sick, or among those facing the dire economic consequences of the pandemic (1–3). In this challenging recovery phase of the pandemic, the mental health needs of healthcare workers and those on the frontlines of the pandemic response cannot be overlooked.

During recent years, the mental health needs of healthcare providers have been gaining attention as a major public health concern and threat to quality care delivery. Healthcare professionals are exposed to multiple stress factors within their work, which may influence their physical, mental, and emotional well-being in negative ways (4–6). The World Health Organization estimates a projected shortfall of 18 million health workers by 2030, mostly in low- and lower-middle income countries. However, countries at all levels of socioeconomic development face, to varying degrees, difficulties in the education, employment, deployment, retention, and performance of their workforce (7). The COVID-19 pandemic is likely to exacerbate these issues among healthcare workers across the globe. In this article we reflect on the mental health impacts on healthcare professionals during times of crisis and other challenging working conditions against a backdrop of the current COVID-19 pandemic. First, we provide a broad overview of the elevated risk of stress, burnout, moral injury, and mental health challenges experienced among health workers. Next, we consider how public health emergencies, such as pandemics, can exacerbate these concerns and pose additional challenges to reaching and supporting health workers. Further, we consider promising approaches for protecting and promoting the mental health of health workers through self-care and other evidence-based interventions. Lastly, we highlight the need for organizational measures, policies, and systemic changes needed to address these challenges and empower healthcare workers going forwards.

## Stress, Burnout, and Mental Health Challenges Among Healthcare Workers

Numerous factors contribute to elevated stress among healthcare workers, including heavy workloads, long shifts, a high pace, lack of physical or psychological safety, chronicity of care, moral conflicts, perceived job security, and workplace related bullying or lack of social support. The resulting psychological distress can lead to burnout, depression, anxiety disorders, sleeping disorders, and other illnesses (5, 6, 8, 9). Work related stress can have a negative impact on health care providers' professionalism, quality of care delivery, efficiency, and overall quality of life. Therefore, it is critical to identify and mitigate these work-related risk factors to protect the mental health and well-being of healthcare workers.

Working in a stressful or challenging environment for long periods with little recovery time is a risk factor for burnout. Burnout is defined as an occupational phenomenon in ICD-11: “Burnout is a syndrome conceptualized as resulting from chronic workplace stress that has not been successfully managed. It is characterized by three dimensions: (1) feelings of energy depletion or exhaustion; (2) increased mental distance from one's job, or feelings of negativism or cynicism related to one's job; and (3) reduced professional efficacy. Burnout refers specifically to phenomena in the occupational context and should not be applied to describe experiences in other areas of life” (10). Maslach et al. describe burnout as that point at which important, meaningful, and challenging work becomes unpleasant, unfulfilling, and meaningless. Energy turns into exhaustion, involvement (also referred to as engagement) becomes cynicism and efficacy is replaced by ineffectiveness (11).

A study investigating burnout and work-life integration in physicians between 2011 and 2017 in the US, found that about 44% of physicians reported at least one symptom of burnout in 2017 compared with about 54% in 2014 and about 45% in 2011 (12). This indicates some fluctuation in physician burnout in the years before the COVID-19 pandemic, yet the levels of burnout among physicians remained significant. Even when adjusting for age, sex, relationship status, and hours worked per week, physicians were found to be at increased risk for burnout and less likely to be satisfied with work-life integration compared with other working US adults (12). Studies have shown that physicians in clinical practice can be at risk for burnout as a result of both work and structural issues. Work related risk factors include work overload (e.g., large patient volumes, insufficient resources, or feeling poorly managed), lack of control over one's work environment, having to spend time on tasks inconsistent with one's career goals and high levels of work-home interference (4, 13). Structural issues predisposing physicians to burnout include being female, working in a solo practice, being early in one's career, lacking a sense of personal control over events, and attributing success to chance instead of personal accomplishments (14, 15). Also, in many low and middle income countries the ratio between healthcare workers and the overall population is a major issue which adds to healthcare workers' work burden, stress, and burnout. Additionally, many frontline health workers in lower income countries are predominantly women, and are therefore typically at the bottom of health system hierarchies, leaving them with limited autonomy and at elevated risk of burnout (16).

Burnout in healthcare workers can also have adverse impacts on patient care. Several cross-sectional studies have linked burnout to suboptimal patient care practices [e.g., (17–20)]—as well as with a doubled risk of medical error and a 17% increased odds of being named in a medical malpractice suit (21). Self-perceived major medical errors were also associated with worsening burnout, depressive symptoms, and a decrease in quality of life. This suggests a bidirectional relationship between medical errors and distress (22). Burnout has also been shown to contribute to a higher risk of motor vehicle accidents (even after adjusting for fatigue) among physicians (23). Other consequences of burnout include absenteeism, low organizational commitment, increased turnover of skilled staff, and greater patient dissatisfaction (11).

Ethical dilemmas [e.g., (24, 25)] and moral injury [e.g., (26, 27)] are other issues that healthcare workers are faced with while providing care within challenging healthcare contexts. Moral injury is defined as the psychological distress that results from actions, or their absence, that violate someone's moral or ethical code (28). The concept of moral injury describes the ambivalence and guilt felt when one's decisions or actions are not in accordance with one's own moral values. It has been characterized as an invisible epidemic among healthcare providers (29). Examples might include where a healthcare worker has to make the difficult decision as to who will get oxygen or be put on a ventilator and who will not if there are a finite number of life saving oxygen points or ventilators available.

Moral injury is not a mental illness, but those who do develop moral damage are likely to see themselves negatively, question their actions and experience feelings of guilt and shame (30). These negative thoughts may contribute to the development of mental illness issues like depression, suicidal ideation and post-traumatic stress disorder (PTSD), as well as thoughts about leaving one's profession (31–35). We could expect increased cases of moral injury when dealing with a health crisis or challenging and stressful working environments; where important decisions—perhaps concerning life and death—are required to be made fast and where the ability to follow optimal care protocols is reduced. Feelings of individual or unclear responsibility is likely to be another risk factor in this context.

The concept of *vicarious traumatization*, also defined as secondary traumatic stress, has also been gaining increased attention during the last decades (36). This condition is associated with different psychological abnormalities derived from the sympathy of healthcare workers toward people who are experiencing primary trauma. Common symptoms associated with vicarious traumatization are loss of appetite, fatigue, irritability, inattention, numbness, sleep disorders, fear, and despair. Frequently, these symptoms are accompanied by trauma responses and interpersonal conflicts. However, the symptoms often remain at subclinical levels (37, 38).

Further, studies have indicated that healthcare practitioners are likely to suffer in silence due to the perceived stigma associated with experiencing “stress” and “mental illness,” as well as fear of getting their medical license withdrawn (39). The stigma accociated with mental health issues has inward-facing impacts for health professionals' willingness to seek help or disclose a mental health problem, which can result in an over-reliance on self-treatment, low peer support—including ostracization and judgment from co-workers if disclosure does occur—and increased risk of suicide (40). A systematic review and meta-analysis of various studies which have been conducted across the world showed high suicide rates among medical professionals across countires, especially among women (41). It also showed that some medical specialties might be at higher risk; such as anesthesiologists, psychiatrists, general practitioners, and general surgeons. There was an overall rate of 1.0% suicide attempts and 17% suicidal ideation among physicians (41). Further, mental health problems, stress, compassion fatigue, and burnout at work are leading causes of healthcare workers thinking about leaving their profession around the world (42–45). When healthcare workers quit, or when they are tragically lost to suicide, they take many years of invaluable training and experience with them. Beyond the individual health workers and their immediate families, this has devastating consequences to their co-workers and to entire health systems, while creating further challenges to meeting the needs of diverse patient groups.

Overall, according to the World Medical Association “physicians in many countries are experiencing great frustration in practicing their profession, whether because of limited resources, government and/or corporate micro-management of healthcare delivery, sensationalist media reports of medical errors and unethical physician conduct, or challenges to their authority and skills by patients and other health care providers” (46). Other subgroups of healthcare workers face similar frustrations and challenges.

## Mental Health of Healthcare Workers in Times of Pandemics and Crisis

During the COVID-19 pandemic, which has been the cause of more than 2.85 million deaths worldwide to date (5th April, 2021) and rising, many healthcare workers, both within medical care and the mental health sector, have been experiencing challenges in adapting quickly to changes in patient volume, mounting demands, clinical roles, new technologies, and ways of working. They have also faced quite high risk of infection together with limitations in protective equipment, as well as managing the anxieties of patients and facing uncertainty in how to effectively treat and respond to complex manifestations of the virus. Perhaps left with no other choice than to adapt as best as they can to ensure the continuation of their obligations and services, many health workers have experienced elevated psychological distress, burnout, and increased risk of mental illness (47). The recovery phase had hardly begun in many countries around the world, when they were faced with a “second wave” or even a “third wave” of the pandemic—and there will probably be several more waves to come as new variants emerge and as we are assessing immunity following vaccination. This means that healthcare providers need to prepare for a continuation of challenging working conditions for quite some time to come. Based on such working conditions and being deployed on the frontlines in the pandemic response, it is perhaps no surprise that issues related to mental health among healthcare workers have drawn interest since the early days of the COVID-19 pandemic (9, 34, 48). Recent surveys, reviews and meta-analyses are pointing to early evidence suggesting that a considerable proportion of healthcare workers have experienced stress, anxiety, depression, and sleep disturbances during the pandemic, raising concerns about risks to mental health (5, 49, 50). Specifically, a recent systematic review and meta-analysis conducted by Li et al. (51) across 65 studies, involving 97,333 health care workers in 21 countries, has identified a high prevalence of moderate depression (21.7%), anxiety (22.1%), and PTSD (21.5%) among healthcare workers during the COVID-19 pandemic. Further, a review evaluated seven studies on COVID-19 related traumatic stress, where five assessed traumatic stress response, one assessed acute stress symptoms and one focused on vicarious traumatization. The findings in all the included studies highlighted the presence of trauma-related stress, with a prevalence ranging from 7.4 to 35%, particularly among women, nurses, frontline workers and in workers who experienced physical symptoms (52). A survey by Li et al. (38), utilizing a mobile phone app-based questionnaire, found evidence of vicarious traumatization in the general public (*n* = 214), frontline nurses (*n* = 234), and non-frontline nurses (*n* = 292) aiding in COVID-19 control during the outbreak in China in February 2020. Another survey conducted among physicians (*n* = 2,334) in the US in August 2020 found that 58 percent of physicians reported to often have feelings of burnout, compared to 40 percent in 2018. Further, nearly 1 in 4 physicians (22 percent) reported to know a physician who has committed suicide, while 26 percent know a physician who has considered suicide. Furthermore, 18 percent of physicians reported to have increased their use of medications, alcohol, or illicit drugs as a result of COVID-19's effects on their practice or employment situation (53).

Vizheh et al. (9) conducted a systematic review of 11 studies assessing health workers' psychological well-being during the COVID-19 pandemic. These studies showed that various factors were associated with mental pressures experienced by the healthcare workers. Working in areas with a high incidence of infection was significantly associated with higher stress and psychological disturbance. Further, sex-aggregated case data collated by the UN show that more than 70% of COVID-19 infections in healthcare workers in the USA, Italy and Spain are in women (54). In an analysis of the results from a survey investigating how gender and race affect the ways in which health professionals experience the pandemic in Brazil, Lotta et al. (55) found that this rate can partly be explained by the absence of necessary resources provided to these healthcare workers: women, and black women in particular, have less access to personal protective equipment and training. Furter, they argue that female healthcare workers worldwide are also facing the downstream effects of their work, including mental health issues, increased physical violence, alternative arrangements for their families so as to not expose them to risk, and physical exhaustion (56).

These types of issues and concerns may be further exacerbated in difficult work environments, such as in impoverished settings and in low-income or middle-income countries where access to personal protective equipment may be severely limited (3, 57). A scoping review was conducted of 51 studies relevant to mental health conditions among healthcare workers during COVID-19, with a focus on low and middle income countries. Symptoms of depression, anxiety, psychological trauma, insomnia and poor sleep quality, workplace burnout and fatigue, and distress were the main adverse mental health outcomes found across these studies. Further, females, younger-aged, frontline workers, and non-physician workers were found to be affected more than other subgroups (58). In a factor analysis study conducted in the early phases of the pandemic, 140 healthcare workers of a tertiary care hospital in India were assessed for perceived stress and insomnia. The factor analysis yielded four factors which were identified as (1) Insomnia, (2) Stress-related Anxiety, (3) Stress-related Irritability, and (4) Stress-related Hopelessness. These four factors explained 62.2% of the variance. Doctors were found to have the highest level of anxiety among all healthcare workers. Both doctors and nurses perceived a greater level of overload related irritability than the other healthcare workers. Compared to doctors and nurses, other healthcare workers were more likely to experience insomnia (59).

The increased negative mental health impact on healthcare providers during times of health crises is not a new concern. Prior reviews have reported on the dire consequences of public health emergencies for frontline health workers, with the most severe impacts often observed in lower-income countries where health systems are fragmented and where there are few protections in place for frontline health workers (16). Several months after the peak of the 2003 SARS outbreak in Hong Kong, healthcare workers reported high levels of ongoing mental distress (60). Studies conducted at the time of the SARS outbreak have also shown that emergency department staff are at higher risk of developing posttraumatic stress disorder (PTSD). Healthcare workers who volunteered in West Africa during the 2013–2016 Ebola epidemic reported symptoms of isolation, depression, stigmatization, interpersonal difficulties, and extreme stress after they returned home (61). About 64% of medical staff working during the initial stages of the MERS outbreak reported PTSD-like symptoms. Staff who performed MERS-related tasks showed the highest risk for post-traumatic stress disorder symptoms even after time had elapsed. The risk increased even after home quarantine (62).

Prior research has reported that disasters have significant adverse effects on the mental well-being of medical responders. Nurses have been found to report higher levels of adverse outcomes, like depression and PTSD, than physicians. Lack of social support and communication, maladaptive coping, and lack of training are important risk factors for developing negative psychological outcomes across different types of disasters (63). Moreover, studies related to emergency situations and disasters, show that long shifts over a longer period of time and risk of personal injury or contamination is associated with an increased risk of sleep disturbances, harmful alcohol use, anxiety, depression, and PTSD among first responders (64–66). These conditions can be broadly similar to those frontline workers are faced with while working with patients with COVID-19 and wearing limited personal protective equipment. These individuals have been putting their own health and safety at risk for long periods of time, striving to help as many people as they can—while working long hours within stressful environments. Sadly, the COVID-19 pandemic is likely to increase healthcare professionals' mental health issues and suicide rates, especially in low-resource settings and countries with few or limited protective measures in place. In the coming months and years, when the “fight or flight” phase of the crisis is over—we will start to see all the different mental health impacts much more clearly. Should history repeat itself, the negative health impacts of COVID-19 on frontline health workers and all types of healthcare professionals are likely to continue for years or even decades to come (67).

While evaluating the emerging research on the mental health impacts of the pandemic, it is important to note that some of the conducted surveys are using screeners which are focused on the immediate effects and which only pick up “diagnostically valid” issues or disorders. However, mental well-being is not just an absence of a mental disorder. Chronic stress, fatigue, fear or guilt of transmitting the infection to loved ones, overwork, self-blame, fear of infection and mortality, lack of breaks or leaves and inflexibility in work schedules can all adversely affect the mental health of healthcare workers—whether they fulfill a set of criterion of a disorder, or not.

Several larger research projects are now in place on national level around the world (e.g., in Norway led by the Norwegian Institute of Public Health) to collect data on both short term and long term mental health impacts on frontline workers and other healthcare staff during the COVID-19 response. As data from such studies starts to emerge, along with the collection of invaluable first-person accounts and qualitative data, the total picture of the COVID-19 related and long-term mental health impacts on healthcare staff will get clearer.

## The Importance of Practicing Self-Care

Most health care professionals are trained to put patients first. Self-care is not always prioritized among clinicians, as they may fear judgment from others or feel selfish at the thought of attending their own needs. Practicing self-care could however be imperative to coping with the obligations, workload, and demands of their profession, and help health professionals gain a better balance or integration between their work and their spare time—as well as help protect their health, well-being, and satisfaction with both their work and overall life.

Research has indicated that effective self-care practices involves self-awareness, self-compassion, the practice of altruism and the implementation of a variety of strategies across physical, social and inner self-care domains (68–70). In a national study of Australian nurses and doctors within palliative care units, 100% of those using a self-care plan reported it to be an effective strategy, while 70% of those not currently using a self-care plan indicated they would consider developing a self-care plan if they were supported to do so (70).

Two recent reviews (71, 72) point to the importance of balancing one's personal needs and the needs of others and recommend self-care as the first line of defense for healthcare workers to manage COVID-19 patient care demands, the longevity of the crisis, and its disruption of normal life routines. In this regard, these reviews highlight the importance of using supportive tools and techniques to combat mental health issues and compassion fatigue among healthcare workers. Some of the suggested preventive self-care strategies include spiritual practices and relaxation techniques, using e-mental health services and enhancing interpersonal skills. Other evidence-based self-care strategies include prioritizing close relationships such as those with family, maintaining a healthy lifestyle by ensuring adequate sleep, regular exercise, and time for vacations, fostering recreational activities and hobbies, and practicing mindfulness and meditation (73).

Practicing self-care is important for all healthcare workers, especially during times of crisis, uncertainty and higher demands. This can be done by frequently checking in with and being aware of one's own emotional level and stress level, taking breaks whenever possible, practicing healthy daily routines like eating healthy food, exercising, or taking walks in nature, getting enough sleep, and allowing emotional processing whenever possible and through the means preferred—whether it is through relaxation, mindfulness and meditation exercises, journaling, taking a run, dancing, engaging in arts or creative work, spending time in nature, calling a friend, or crying in solitude. Allowing emotional expression on a continuous basis—as opposed to repressing emotions—helps build emotional resilience and mental well-being in the long run (74). The beneficial effects of appropriate self-care for healthcare workers include improved physical, mental, and emotional well-being (75), as well as being able to provide care for their patients in a more sustainable way with greater compassion, sensitivity, effectiveness, and empathy (76). Practicing self-care can also help healthcare workers create some structure and predictability amidst chaos and uncertainty and make them able to manage high levels of stress in more constructive ways. A qualitative study based on semi-structured interviews with 172 physicians working in COVID-designated centers across India, found support for resilience as a learned and evolving process: Most of the participants mentioned resilience as a continuum developed through experiencing and facing an unprecedented crisis, aided by social support and past encounters with stress. The results suggested that the consistent living through hardships and adversities of the COVID-19 crisis with responsible risk-taking, helped pave the way for problem-solving, personal efficiency and coping in the physicians (77). Empathy, optimism, and self-efficacy can also improve personal health-risk perception, which is vital for psychological resilience during pandemics (77, 78).

The recovery phase is when the healthcare workers will have more time for reflection, contemplation and rumination around the experiences they have been through during a crisis. It is essential for their recovery that they have professional support and appropriate mental health and self-care tools—and knowledge about how to use them—during this phase. Especially when the time of recovery is short, and interrupted with new waves of virus outbreaks or other challenging crises. It's also essential that healthcare providers focus on their strengths, instead of engaging in self-critical thinking about things they could have done better. To avoid accumulation of stress during times of crisis or other challenging working conditions, healthcare workers should try to prioritize and simplify tasks; focus on one task at a time whenever possible; set healthy boundaries; communicate in a self-assertive way; and seek support for important clinical decisions. For some, practicing self-care and being able to debrief with colleagues will be enough, while for others who might be traumatized or who experience high levels or stress, anxiety or depression, stronger measures, and opportunities for professional support over time will be needed.

## Proactive Prevention Measures and Interventions

Short-term mood boosters have been widely practiced during the COVID-19 pandemic. These have included free lunches or snacks during the working day, or clapping, posters, and songs thanking “healthcare heroes” for their efforts. While this may offer temporary acknowledgment, it can also seem like a distraction or excuse from attending to the serious challenges frontline workers and other health professionals face in regards to protecting their own health and well-being at work (79). In fact, repeatedly referring to healthcare workers as “heroes” may actually act as a barrier to them seeking help (i.e., heroes help others; they don't need help). Healthcare workers need to be seen as fellow human beings who are not invincible and whose resilience also has limits. Instead of superficial or temporary appraisal and reward, we need to invest in and accelerate protective and preventive measures to reduce the burden on healthcare workers on a more permanent basis. This should also involve leveraging existing evidence-based interventions for alleviating psychological distress in public health emergencies.

As we have seen, healthcare providers who have worked on the frontline, humanitarian aid workers, lay providers, as well as mental health professionals providing psychological first-aid and mental health care for those affected during these times of crisis, are all exposed to, and can be expected to experience significant negative health impacts like post-traumatic stress, anxiety, insomnia, and depression. As mentioned, we also know that physicians and other healthcare providers may be hesitant to seek mental healthcare, often due to concerns about confidentiality and its potential impact on their careers (39).

For too long, the responsibility has been on the individual healthcare worker to recognize and manage their own stress, burnout or depression, with few avenues or tools made available for them to successfully do so. COVID-19 has clearly demonstrated the need to invest in protecting the mental health of the healthcare workforce. Based on the current situation and working conditions for healthcare practitioners, as well as earlier research findings on the negative mental health impacts from emergency situations and the difficult and stressful working conditions in general, it should be of highest priority to prevent a parallel pandemic of mental health issues among the healthcare workforce in the times to come.

Thus, it is imperative to provide necessary mental health support for healthcare staff during these times. Healthcare workers across the medical and mental health sector should be offered psychological first aid during times of crisis, heavy workload, or challenging working conditions, as well as long-term support through accessible mental health support programs (e.g., resilience, self-care, or mindfulness courses). For instance, there are brief psychological interventions that have proven to be effective in managing stress in humanitarian emergencies. The World Health Organization's Self-Help Plus (SH+) stress management intervention (80) may be ideal for addressing the elevated psychological stress and risks of burnout and mental distress among healthcare workers responding to the COVID-19 pandemic. SH+ is based on acceptance and commitment therapy (ACT), which can reduce symptoms of depression and anxiety (81), and combines elements of cognitive behavioral therapy (CBT) including psychoeducation, mindfulness exercises, and promoting psychological flexibility where individuals learn new ways to open up to and cope with difficult thoughts and feelings consistent with their own values, rather than avoiding these thoughts (82). One key feature of SH+ is the broad focus on reducing psychological distress associated with adversity and difficult life experiences (80), which increases scalability by not requiring use of diagnostic procedures or targeting specific syndromes. It is important to recognize that even though health workers during the pandemic are experiencing high rates of distress, this does not mean they have a diagnosable mental disorder, emphasizing the need for early intervention and self-help in alleviating stress. SH+ has demonstrated effectiveness in significantly reducing psychological stress among women in humanitarian settings in Uganda, as reflected in a cluster-randomized trial with 14 villages enrolling 694 participants (83).

The use of telemedicine centers, platforms, and solutions is a promising strategy to provide more effective care for patients, while reducing the workload and taking off pressure on healthcare workers. During the COVID-19 outbreak in China there have been positive experiences with using remote consultations to connect medical experts in a telemedicine center with frontline health workers in the Hubei province (and other less developed areas) to share medical images, record data, and test results. A telemedicine platform was also set up where medical workers could share their opinions and experiences with each other in order to achieve more suitable treatment methods. The platform was also used to provide psychological counseling for frontline doctors through videoconferencing to help them cope with stress and anxiety. This was found to greatly reduce the pressure on frontline medical staff. Other potential benefits with this approach are reduced needs for face-to-face consultations, protection of experts from exposure to viruses and saved costs (84). Thus, telemedicine approaches can help prevent work overload, burnout, and mitigate negative mental health impacts in healthcare workers in real time.

Mindfulness practice and stress management approaches are two additional health interventions with evidence of efficacy and measurable outcomes for reducing burnout and promoting resilience in clinicians (85, 86). Maunder et al. (87) found that computer-assisted resilience training in healthcare workers appeared to be of significant benefit under pandemic conditions. Peer-to-peer support groups and buddy teams are other helpful interventions that could give healthcare workers a possibility to debrief their experiences with others with similar experiences (30). The use of reflective practice groups or “reflective rounds” (e.g., Balint, Schwartz) have a growing evidence base in helping clinicians manage stressful encounters and in reducing the impact of burnout. For example, eight face-to-face reflective rounds were arranged for staff in Southampton Children's Hospital in the UK, from September 2017 to February 2020 with a further virtual round in July 2020 during the COVID-19 pandemic. Each round was facilitated by a clinical psychologist and consultant. For each round, up to three volunteer panelists from different staff groups were invited to share their personal experiences on a pre-selected subject to the large group. The group would then contribute to the discussion by offering their own reflections. Feedback on this practice was received from 202 participants. The results showed that the majority (98%) would recommend the rounds to colleagues with 64 participants (32%) rating the rounds as “exceptional” and 91 (45%) as “excellent.” The virtual round received similar positive feedback (88).

Such evidence-based prevention measures and interventions will help protect and strengthen the mental health and resilience of healthcare workers in the long run, and make them better prepared to manage and thrive in their work during challenging circumstances. It will also help facilitate their personal and professional development on an ongoing basis.

## Workplace Culture and Leadership

Unfortunately, no measures to prevent burnout or other mental health issues will be effective unless attention is paid to enhancing a positive work environment, defined as one “that attracts individuals into the health profession, encourages them to remain in the health workforce and enables them to perform effectively to facilitate better adaptation” (89). Healthcare leaders and decision makers should seek to lead by example and work toward reducing the stigma associated with mental health issues among healthcare staff, and foster a work culture of transparency, trust, respect, openness, equality, empathy, and support. Healthcare leaders should especially be mindful of promoting a culture of inclusion, collaboration, and support, instead of comparison and competition.

It is crucial that healthcare leaders and decision makers step up and take long-term responsibility when it comes to highlighting the importance of protecting the mental health of the healthcare workforce on an ongoing basis. Normalizing discussions about mental health among physicians and other healthcare workers can help reduce some of this stigma, while establishing long term screening and prevention programs will be critical to monitoring early signs and preventing burnout, PTSD and other mental health issues on a continuous basis. Since the risk of moral injury can be high when faced with a heavy workload, stressful environments, limited equipment or difficult choices, especially during times of public health emergencies, it is a topic worth more attention and interventions from the leadership of health and care organizations. Managers can help staff make sense of morally challenging decisions and their psychological response to these difficult decisions by being straightforward and honest about possible ethical dilemmas. Also, support from colleagues and line managers helps to protect the mental health of healthcare workers in these regards (90).

A study involving a total of 214 healthcare workers in Turkey found that in order to raise the psychological resilience of healthcare professionals working during the COVID-19 pandemic their quality of sleep, positive emotions, and life satisfaction need to be enhanced. The study also concludes that in order to enhance positive emotions and weaken negative emotions of healthcare professionals, the workers' needs ought to be prioritized in any practice (91). Rieckert et al. (92) conducted a scoping review of 73 papers focusing on the impact of COVID-19-like working conditions on the physical and/or mental health of healthcare professionals in a hospital setting, as well as on interventions, measures and policies to preserve physical and/or mental health. The review was supplemented with expert interviews to validate the findings. The results showed that recommendations fostering resilience prior to the outbreak included optimal provision of education and training, resilience training, and interventions to create a feeling of being prepared. Recommendations during the outbreak consisted of (1) enhancing resilience by proper provision of information, psychosocial support, and treatment (e.g., create enabling conditions such as forming a psychosocial support team), monitoring the health status of professionals and using various forms and content of psychosocial support (e.g., encouraging peer support, sharing, and celebrating successes), (2) tasks and responsibilities, in which attention should be paid to kind of tasks, task mix, and responsibilities as well as the intensity and weight of these tasks and (3) work patterns and working conditions.

In line with such findings, healthcare staff should be given the opportunities to express their needs and the means to have these needs met. Further, turning personalized self-care plans and approaches to build resilience into standard organizational cultural practice is important in assisting with overcoming barriers and prioritizing self-care among healthcare workers. At an organizational level, time to and ventures of practicing self-care should be recognized as a needed and essential part, both in terms of preventing mental health issues and building resilience and for increasing motivation, job satisfaction, patient safety, and quality of care. The self-care, resilience, and workplace policy interventions which are promoted on an organizational level could involve mandatory breaks (including power naps, taking walks, access to silent rooms, etc), use of mini-breaks before every meeting, policies to have all meetings to start on the hour and other approaches to improving the workplace environment.

Also, a limit on the length of work shifts should be put in place, and there should be opportunities to enroll in professional mental health support programs and to engage in peer-to-peer support groups, reflective rounds, or buddy teams to debrief their experiences on a regular basis. Furthermore, hospitals and other care facilities should establish adequate parental and sick leave policies that do not burden a healthcare provider's colleagues. Departments should schedule in protected time for personal doctor, therapist, and dentist appointments. Other aspects of policy change likely to have a positive impact on the well-being of healthcare professionals is in relation to non-punitive responses to medical errors (93) and a trauma-informed approach to stress in the workplace. In addition to common challenges and needs which are necessary to address within the healthcare workforce as a whole, the reviewed literature has also pointed toward heterogeneous needs among healthcare workers based on specific challenges, predispositions, socio-economic factors, gender, race, and other factors. It is important that healthcare leaders and decision makers are aware of such differences in needs, and that their policies and interventions help reduce the extra burdens on those who are most vulnerable to experience adverse mental health impacts as a result of their working conditions.

Essentially, protecting the mental health and the professional and personal development of healthcare staff, should be a high priority not only in times of crisis, but on an ongoing basis. A recent report from the National Academy of Medicine in the US (2019) recommended that medical societies, state licensing boards, specialty certification boards and medical education and health care delivery organizations all need to take concrete steps to reduce the stigma for clinicians of seeking help for psychological distress and make assistance more easily available. These recommendations are even more relevant during the COVID-19 pandemic.

## Towards a Systemic Shift

A survey conducted by the World Health Organization on the impact of COVID-19 on mental, neurological, and substance use services in 130 countries, clearly indicates that mental health systems have been compromised at a time when they are likely to be needed the most (94). Healthcare systems should be reimagined to reduce the increasing burden on healthcare professionals, and promote their health, well-being and thus their ability to provide care and achieve optimal outcomes. We need healthcare systems that are driven by the needs of patients and healthcare providers; and not by demands of increased efficiency and economic interests only. This change should be reflected by policies, professional guidelines or other means of self-regulation.

A recent policy brief by United Nations on COVID-19 and the need for action on mental health suggests that the implementation of the following systemic actions by national decision-makers will help minimize and address the mental health consequences of the pandemic in general: (1) Apply a whole-of-society approach to promote, protect and care for mental health, (2) ensure widespread availability of emergency mental health and psychosocial support, and (3) support recovery from COVID-19 by building mental health services for the future (95).

Furthermore, involving both healthcare providers and patients in evaluations and improvements of healthcare services, so called *co-production* (96, 97), can help share power (98) and provide valuable insights on both individual and organizational level. Exercising the principles of co-production, healthcare workers should be involved in the process of decision making, development, implementation, testing, and evaluation of interventions and efforts aimed at preventing psychological distress and mental health issues and improving their health, well-being, and job satisfaction in the long run. Further, including feedback from both patients and healthcare providers about perceived quality of care and working conditions, may also result in other important organizational benefits like financial support for improvements from higher management bodies such as the hospital's medical staff and Board of Directors. Such input can then be used as an instrument to gain legitimacy and set necessary improvements in motion (99). Exercising the concept of co-production within healthcare systems, could therefore help improve both the quality of care and the working conditions and well-being for overworked health care providers.

Similarly, it is important to address stigmatization within healthcare facilities as a systemic issue and keep those who fear or are being burdened by stigmatization at the center of any response to stigma. This includes working to empower people or groups experiencing stigma (including patients, caregivers, and healthcare workers), for example, by building skills and efficacy to address internalized stigma and cope with and challenge stigma, as well as building partnerships with gatekeepers and opinion leaders for change (100). Focused social and organizational support and understanding of the distress faced by healthcare workers during times of crisis and in general, can help reduce the stigma and improve social connections while reducing barriers to utilization of mental health interventions aimed at supporting healthcare workers. This has been termed an “epidemic of empathy” and has the potential of bringing together science and humanism in a way that might be beneficial even after cessation of the pandemic (77, 101).

Finally, there is a need for prioritizing and promoting global collaboration on research, including longitudinal and qualitative studies, on the mental health impacts on healthcare workers during the pandemic and in the post-pandemic aftermath. The voices and first-person accounts of frontline workers and other healthcare staff who have served during the COVID-19 pandemic across the globe will bring invaluable contributions to the field and help foster needed systemic changes, rather than relying on quantitative studies and mere statistics or survey studies alone.

Implementing and promoting such systemic changes will be especially important in low and middle-income countries, where the vast majority of the world's population reside; yet, access to effective mental health care services remains severely constrained. It is in these lower resource settings where advances in the delivery of mental health care will be especially impactful at a population level, by advocating for the inclusion of these services as part of efforts to achieve universal health coverage, and more specifically, by recognizing the needs of healthcare providers delivering mental health services to vulnerable population groups across diverse settings.

## Policy Recommendations

Based on the findings in the reviewed research and the points raised in our discussion, we propose the following policy recommendations:

- Enact national and local evidence based interventions and programs to support frontline healthcare workers health and well-being in a long-term perspective. Leverage the necessary expertise of health, wellness and behavioral science experts to guide the implementation of these solutions and ensure clear evaluation design, analysis, and iteration to inform continual evaluation and improvement.
- Share and distribute these resources across the organization, network of partners, patients, and others.
- Create national knowledge-bases (information, tools, and resources) designed to improve the resilience and well-being needs of workers and their leaders in times of crisis, recovery, and rebuilding.
- Ensure adequate staffing levels in healthcare systems and fair pay for workers.
- Encourage help-seeking and ensure available mental health resources for frontline health workers in distress.
- Condemn and combat the stigmatization of frontline healthcare workers. Increase efforts to de-stigmatize mental health across society.
- Ensure a wider and more actionable dialogue about mental health in the workplace.
- Engage frontline healthcare workers in the political decision-making processes and in co-creating new policy development.
- Reallocate research funds to explore paths for future preparedness for frontline healthcare workers.
- Consider the opportunity for digital technology and other innovative approaches to ensure access to effective training and ongoing support and guidance among frontline workers and in the overall healthcare workforce.

## Conclusions

Healthcare workers across health systems and disciplines are facing significant stressors, burdens, and mental health challenges as a result of their work. This is especially the case for those who work on the frontlines during public health emergencies—with further challenges faced by those who work in impoverished and low-resource settings or in settings where stigmatization is high. The COVID-19 pandemic has acutely reminded us of the important and invaluable work that frontline workers and other healthcare professionals do on a daily basis in challenging circumstances, and has exposed the limitations of healthcare systems around the world. Before the memory of the pandemic response starts to fade, appropriate evidence-based measures and interventions must be put in place and actioned to protect the mental health and well-being of the healthcare workforce—not only during public health crises, but on a day-to-day basis. The measures and policy recommendations outlined in this article are a few of the many meaningful interventions that can reduce the risk of healthcare providers incurring ongoing, long-term psychological damage in the wake of COVID-19 and beyond.

Healthcare workers should be respected for the vital work they do to keep populations healthy, meaning we have a duty to find ways to meet their psychological needs and improve their welfare. The authors are hopeful that the acknowledgment and appreciation of the healthcare workforce will continue and become more permanent in the times to come. Empathy, transparency, open disclosure, and effective and supportive communication will solidify the partnership and collaboration between healthcare leaders, healthcare providers and patients as well as other stakeholders. This will then in turn provide the foundation of a healthcare system that revolves around the improvement of experiences and well-being outcomes of all involved.

World leaders and other decision makers need to fully realize the crucial importance and value of investing in the mental health and well-being of the healthcare workforce, on individual, organizational, and societal level. As well as the personal, economical, and societal benefits of doing so. Let us learn from our past and thank our essential healthcare workers by demanding and promoting real reforms within our healthcare systems. We cannot afford the cost of failing in this aim.

## Data Availability Statement

The original contributions presented in the study are included in the article/supplementary material, further inquiries can be directed to the corresponding author/s.

## Author Contributions

LS conceived the main conceptual ideas, drafted the manuscript, and performed the main literature review as well as subsequent editing and corrections. JN contributed with a critical review, helped drafting the introduction section, and contributed with additions, literature citations, and edits to the different sub sections of the manuscript. AK contributed with a critical review, literature citations, and edits in different sub sections of the manuscript. SS contributed with a critical review, literature citations, and edits in specific sub sections of the manuscript. MWQ provided early support for the main conceptual ideas and contributed with literature citations and input for different sub sections of the manuscript. CG contributed with literature citations and edits to specific sections of the manuscript. LM contributed with comments and suggestions to specific sub sections of the manuscript. All authors contributed to the article and approved the submitted version.

## Conflict of Interest

MWQ was affiliated with the company 21HealthStreet. The remaining authors declare that the research was conducted in the absence of any commercial or financial relationships that could be construed as a potential conflict of interest.
